# Peptide Nucleic Acids and Gene Editing: Perspectives on Structure and Repair

**DOI:** 10.3390/molecules25030735

**Published:** 2020-02-08

**Authors:** Nicholas G. Economos, Stanley Oyaghire, Elias Quijano, Adele S. Ricciardi, W. Mark Saltzman, Peter M. Glazer

**Affiliations:** 1 Department of Genetics, Yale University School of Medicine, New Haven, CT 06520, USA; nicholas.economos@yale.edu (N.G.E.); elias.quijano@yale.edu (E.Q.); 2 Department of Therapeutic Radiology, Yale University School of Medicine, New Haven, CT 06520, USA; stanley.oyaghire@yale.edu; 3 Department of Biomedical Engineering, Yale University, New Haven, CT 06511, USA; adele.ricciardi@yale.edu (A.S.R.); Mark.saltzman@yale.edu (W.M.S.)

**Keywords:** peptide nucleic acids, PNA, triplex, gene editing, structure, recombination, repair, nanoparticles, β-thalassemia, cystic fibrosis

## Abstract

Unusual nucleic acid structures are salient triggers of endogenous repair and can occur in sequence-specific contexts. Peptide nucleic acids (PNAs) rely on these principles to achieve non-enzymatic gene editing. By forming high-affinity heterotriplex structures within the genome, PNAs have been used to correct multiple human disease-relevant mutations with low off-target effects. Advances in molecular design, chemical modification, and delivery have enabled systemic in vivo application of PNAs resulting in detectable editing in preclinical mouse models. In a model of β-thalassemia, treated animals demonstrated clinically relevant protein restoration and disease phenotype amelioration, suggesting a potential for curative therapeutic application of PNAs to monogenic disorders. This review discusses the rationale and advances of PNA technologies and their application to gene editing with an emphasis on structural biochemistry and repair.

## 1. Introduction

Nucleic acid molecules are capable of forming a broad range of structures due to their backbone flexibility, hydrogen bonding combinations, and sequential monomeric building blocks. While canonically DNA is known to prefer a right-handed double helical structure, or B-DNA, a multitude of alternative nucleic acid structures have been reported since Watson and Crick first described the double helix in 1953 [1]. Left-handed double helical DNA (Z-DNA), triplex DNA (H-DNA), tetraplex DNA (e.g., G-quadruplex), and DNA:RNA looping hybrids (R-loops) are a few examples of the diverse formations these molecules adopt in nature, often in sequence-specific contexts [2]. The consequences of these curious structures have been under investigation for decades revealing critical roles in gene regulation, telomere protection, and recombination processes amongst others. However, while unusual structures expand and nuance the genomic toolbox, these sites have been shown to be associated with genomic instability in some contexts [3]. Consequentially, a suite of dedicated helicases, polymerases, and repair networks evolved in tandem to recognize and resolve these transient structures faithfully and maintain genomic integrity [4]. Unsurprisingly, owing to the inherent sequence-specific, recombinogenic properties of non-B-DNA structures and their ability to elicit endogenous repair, harnessing these molecular configurations as an approach to targeted gene modification has attracted considerable interest [2].

**Figure 1:**
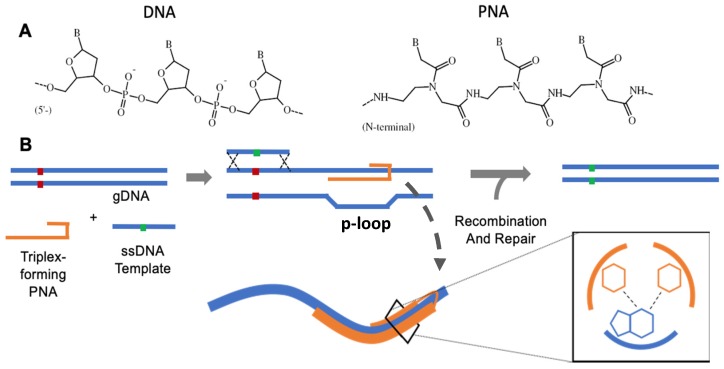
(**A**) Phosphodiester and polyamide backbone structures of DNA and PNA polymers, (**B**) Simplified schematic of triplex-forming PNA-mediated gene editing.

The field of structure-mediated gene targeting developed rapidly as investigators initially pursued the application of triplex-forming oligonucleotides (TFOs)—single-stranded DNA molecules that associate with the major groove of a DNA helix at polypurine stretches and stabilize via Hoogsteen hydrogen bonding (H-bonding) [5]. These sequence-targeted molecules were used to deliver mutagens to induce gene knockout and eventually were applied with short DNA templates to achieve homologous recombination [6,7]. While DNA TFO-induced gene editing demonstrated a modest ability to accomplish targeted sequence modification, the field took a major leap forward with the advent and application of a new class of synthetic DNA prototype, peptide nucleic acids (or PNAs), which are the focus of this review. First described by Nielsen and colleagues in 1991, PNAs are synthetic DNA analogues that feature a polyamide (i.e., protein-like) backbone, as opposed to a conventional phosphodiester backbone (Figure 1A, [8]). This key chemical modification endows PNAs with advantageous characteristics that make them especially amenable to targeted structure-induced recombination. Firstly, as a result of a neutrally charged polyamide backbone, and by minimizing repulsive negative forces between polymer backbones, PNAs are capable of forming remarkably high-affinity base-paired structures with DNA [9]. This effect is even more pronounced when two PNA strands coordinate with a single strand of DNA, resulting in an exceptionally stable heterotriplex structure relative to DNA:DNA:DNA homotriplexes (e.g., TFO) [10]. In contrast to TFOs that bind along the exposed major groove of a target helix, PNAs are capable of invading helices to coordinate around a single strand of DNA while displacing the opposite strand (termed a p-loop, Figure 1B, [5,11]). Further, due to their novel structure, PNAs are resistant to nuclease and protease-mediated degradation and are thus very stable in living cells [12]. Taken together, these attributes allow investigators to wield PNAs as a potent means to generate salient sequence-specific structures with genomic DNA (gDNA). The resulting bulky helix-distorting p-loop robustly recruits endogenous repair and, when provided a co-delivered ssDNA template specifying a sequence change, mediates the incorporation of a persistent genomic edit (Figure 1B, [13,14,15]).

An exception in a field dominated by enzymatic approaches, PNAs are a powerful gene editing technology that utilizes modifiable molecules to generate recombinogenic structures. Decades of advances in PNA chemistries, systems of delivery, and their applications to gene editing have effectively developed this technology into a promising candidate for genetic therapy for systemic monogenetic human disease. Work from our laboratories at Yale University has shown that PNAs can be co-delivered with a ssDNA template using biodegradable polymeric nanoparticles and administered to animals directly in vivo [16,17,18,19,20]. To date, nanoparticle-delivered PNAs have been used to correct disease-implicated mutation in multiple tissues, across multiple disease models in mice—ex vivo, in vivo, and in utero [16,17,18,19,20,21,22]. This review explores advances in PNA gene editing as they relate to PNA chemistry, structural biology, delivery, and repair and recombination before summarizing on-going work and outlooks for this exciting developing field.

## 2. Pre-PNA: TFOs and Structure-Induced Recombination

Early on, investigators identified the potential use of TFOs as a means to recognize unique genomic sequences and to elicit a site-directed effect. Initial experiments from our group (1993) used TFOs to deliver conjugated mutagens, such as psoralen, to induce gene knockout [23]. That same year Kohwi and Panchenko made the fascinating observation that triplex-forming sequences were associated with recombination events in an orientation and length-dependent manner using plasmid-based assays in *E. coli* [24]. Three years later, Faruqi et al. (1996) [25] went on to demonstrate recombination induced by triplex formation via TFO, both with and without psoralen conjugates, in mammalian cells. This advance set the stage for using triplex technologies to induce specified sequence modifications.

In 1999, Chan et al. demonstrated editing at a single base via template-directed recombination for the first time using short ssDNA donor molecules tethered to a TFO targeting an SV40 vector. Editing frequencies occurred in the range of ~0.1% [7]. Later, work from Datta et al. using cell-free extracts showed that the covalent linkage between donor and TFO was not a requirement for editing to occur [26]. Importantly, this result suggested that TFO binding and triplex formation was the key event leading to repair and recombination. This led the authors to believe that strand breaks secondary to triplex repair could be responsible for forming recombination intermediates.

Although triplex editing via TFOs proved modestly effective, a few key limitations hindered overall efficiency. First, early work demonstrated that TFO binding affinity for its target is highly correlated with intracellular activity. Recombination experiments in cell-free extracts revealed that generally TFOs with a *K_D_* (equilibrium dissociation constant) less than or in the range of 10^−7^ M were necessary for measurable activity [27]. *K_D_* was also shown to correlate closely with TFO length, often requiring up to 30 nucleotides for activity, restricting the number of possible genomic targets. Further, because TFOs are composed of DNA they are subject to nuclease-mediated degradation and only transiently remain within the cell intact [12]. Various strategies have been tried in order to surmount some of these restrictions (summarized in sections of [28]), but none have progressed the technology close to therapeutically meaningful efficiencies. While work using TFOs demonstrated a proof-of-principle for using site-specific structure formation to induce recombination, their feasibility as a potentially therapeutic biotechnology fell short. Ideally such a technology needs to demonstrate superior triplex binding kinetics, intracellular stability, and broader versatility.

## 3. PNA Chemistry

The unique features of PNA oligomers satisfy the aforementioned criteria. For example, relatively short (10 mer) PNAs bind with nanomolar affinity (*K_D_* ~ 10^−9^) to complementary DNA targets, with apposite modifications (vide infra) able to improve affinity several orders of magnitude to high femtomolar (*K_D_* ~ 10^−12^) [29]. Further, the bifurcated character of PNAs—whereby the same compound is part nucleic acid, part peptide, but never entirely either—imparts stealth, since no known endogenous nucleases and/or proteases recognize PNAs as substrates [12]. Also, the ease of synthesis, based largely on established protocols for peptide syntheses, and availability of synthetically amenable sites in the backbone have allowed variations in PNA design and conformation to optimize DNA binding efficacy [30]. 

**Figure 2:**
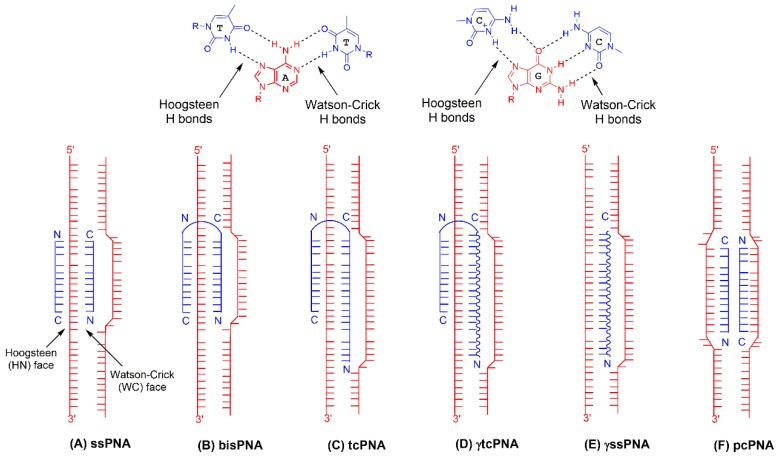
PNA structural variations to drive exergonic strand invasion. (**A**) single-stranded (monomeric) PNA; (**B**) bis (dimeric) PNA; (**C**) tcPNA; (**D**) γtcPNA; (**E**) γssPNA; (**F**) pcPNA.

Although the superiority of appropriately designed PNA oligomers over TFOs for DNA binding is now well established in the literature, these trends are only intuitive in retrospect. In fact, the seminal work introducing PNAs for DNA recognition presented them as ligands for the accessible major groove of purine-rich DNA targets (like TFOs), where they could bind using the same Hoogsteen H-bonding interactions that stabilize TFO-based triplexes [8]. However, this study led to the surprising finding that pyrimidine-rich PNAs formed stable PNA_2_-DNA triplexes on the bound DNA strand by simultaneous displacement of the homologous region of the non-target (pyrimidine-rich) DNA strand [8]. Evidently, the PNA ligand was sufficiently homomorphous to DNA and possessed the binding affinity to invade an otherwise stable duplex structure harboring a complementary sequence, resulting in a complex where two PNA molecules engaged the Watson–Crick (WC) and Hoogsteen (HN) faces, respectively, of the same target strand (Figure 2A [8]). Confirmatory evidence [31] for this binding mode has since incentivized synthetic strategies to optimize the binding efficacy of PNA oligomers for duplex DNA targets, since invasion of B-form DNA at near-physiologic osmolality and acidity has been recognized, then [32] and now [33], as a major challenge for PNA binding. Indeed, it is the evolution of the PNA compound in pursuit of more favorable binding that has largely enabled strategies for PNA-induced gene editing [34].

## 4. Triplex-Forming PNAs for Gene Editing

### 4.1. Bis-PNAs and Early Applications

Because the PNA-DNA binding reaction, like every molecular reaction, is driven by the standard free energy relationship

(1) [ΔG=ΔH−TΔS]

the synthetic strategies to enhance DNA recognition have focused on modulating both terms of the equation (ΔS and ΔH, for entropy and enthalpy changes, respectively) to achieve exergonic (ΔG<0) DNA binding reactions. For example, the realization that two polypyrimidine PNA molecules dimerized on the target DNA strand upon binding [31] led to the initial modification of tethering single PNA strands to create dimeric (bis) PNAs (Figure 2B [35,36]). This strategy effectively converted the binding reaction from a trimolecular process, where two PNA molecules dimerize on the bound DNA, to a bimolecular process, where tethered PNA domains on the same PNA molecule form H-bonds with nucleobase H-bond donors and acceptors on both faces of the target DNA, thus decreasing the translation entropy change (ΔS) required for DNA binding [35,36].

**Figure 3:**
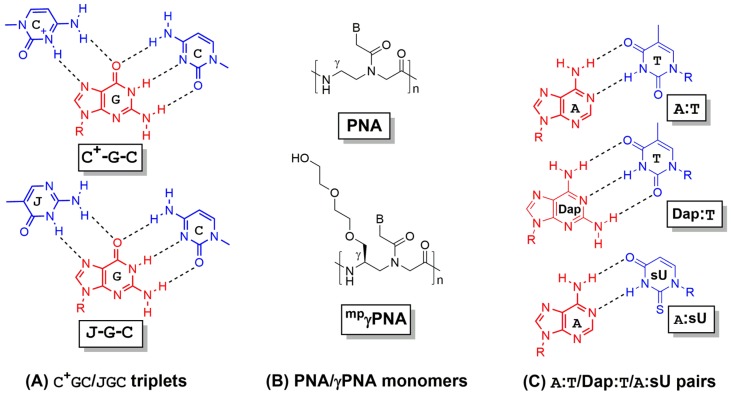
Structural modifications in PNA backbone and nucleobases to enhance strand invasion. (**A**) Hydrogen bonding of C^+^GC and JGC triplets (**B**) PNA and gamma(γ) modified PNA monomers (**C**) A:T, Dap:T, and A:sU hydrogen binding pairs

This new construct was also informed by ancillary studies establishing the orientational preferences for PNA binding to the WC and HN faces of the target strand [37]. Initial reports demonstrated the efficacy of this modification for duplex DNA invasion, with one report showing the EC_50_, PNA concentration necessary for binding 50% of duplex targets, to be > 500-fold higher for a bisPNA relative to the corresponding single PNA [35]. Further, because Hoogsteen base pairing requires the incoming cytosine (C) residues to provide the H-bond donors (Figure 3A), DNA strand invasion by single or bisPNAs, or indeed any PNA variation requiring Hoogsteen pairing, shows a strict pH dependence [38], since p*K*a < pH for C under physiologic conditions [39], requiring acidic conditions for strand invasion. Therefore, the pseudoisocytosine (J) residue, a structural isomer of C that mimics its protonated form (Figure 3A), was introduced to facilitate pH-independent binding [36]. Importantly, this modification allowed detection of DNA strand invasion at bisPNA concentrations three-fold lower than those observed for unmodified bisPNAs [36].

Triplex formation on the bound DNA strand is also accelerated for bisPNAs relative to single pyrimidine PNAs, since hybridization on the WC face of the target, proposed to be the nucleation step for triplex formation, increases the effective concentration of the second, tethered PNA strand for subsequent base pairing on the HN face. This binding model is supported by thermal denaturation data which show reduced hysteresis—variance between the transition profiles recorded for denaturation (melting) and renaturation (annealing), due to slow association kinetics—for bisPNA-DNA complexes relative to those with single PNAs [36]. Because of the inverse relationship between $K_{D}$ and association rate constant $k_{a}$,

(2) $$\left[K_{D}=\frac{k_{d}}{k_{a}}\right]$$

and because of the direct relationship between $K_{D}$ and free energy change,

(3) $$\left[\Delta G=RTlnK_{D}\right]$$

accelerated binding kinetics as enabled by bisPNAs improve the binding efficacy of the ligand for DNA strand invasion by lowering ΔG.

With highly stable triplex formation capability, optimized nucleobase isomers, and dramatically improved binding kinetics, bisPNAs were the first PNAs successfully used for gene editing applications. In 2002, Rogers et al., showed targeted recombination with ssDNA donors using a *supFG1* plasmid reporter system in human cell-free extracts [15]. A single base template-directed base conversion (G to C) in this plasmid system, followed by transformation into bacteria and plating in the presence of β-galactosidase, allowed for the ability to detect rare recombination events. bisPNAs tethered to short DNA templates and unconjugated bisPNAs were tested in this system. Unconjugated bisPNA and donors stimulated recombination at a frequency of 0.08%, an effect fivefold more active that the introduction of donor DNA alone [15].

Chin et al. (2008) went on to demonstrate the effectiveness of bisPNA mediated gene editing in mammalian cell culture using a disease-relevant reporter model [40]. bisPNAs were used to edit a single base β-thalassemia splicing mutation in a human β-globin intron (IVS2-1^G^^→A^) placed within a single copy of the GFP gene. This reporter cassette was placed within the genome of Chinese hamster ovary (CHO) cells. Thus, following a targeted editing event and splicing restoration, appropriately edited cells were able to express full-length GFP mRNA transcripts and fluoresce. In this study, bisPNAs designed to target a homopurine region 193 bp downstream from the target edit and a 60 mer ssDNA donor were co-nucleofected into CHO reporter cells and restored GFP expression in 0.2% of cells [40]. Importantly, GFP expression was maintained in culture for a month after nucleofection suggesting that editing events were persistent, heritable, and occurred as a result of genomic sequence change. Beyond an artificial reporter construct in CHO cells, Chin et al. went on to demonstrate bisPNA-mediated editing in the β-globin IVS2 introns in human K562 cell lines, mouse bone marrow containing a human β-globin locus, and human CD34+ progenitor cells [40]. Notably, after continued culturing, edited CD34+ lines differentiated into myeloid and erythroid lineages with persistent detectable sequence modification. These results highlighted PNA-mediated editing as a feasible translational technology. No longer a conceptual practice in plasmid assays, PNAs were used to genetically manipulate human hematopoietic stem cells (HSCs) ex vivo. This approach, followed by stem cell transplantation, is a viable therapeutic means to treat monogenetic hematologic disorders.

Two additional studies demonstrated the ability of bisPNAs to edit human CD34+ cells in culture. McNeer et al. (2011) employed a novel delivery approach to target CD34+ cells and edit the β-globin IVS2 intron using bisPNAs [41]. By encapsulating bisPNAs and ssDNA donors in spherical poly(lactic-co-glycolic acid) (PLGA) nanoparticles (NP) they showed superior reagent delivery and editing efficiencies approaching 1%. Moreover, using PLGA NPs showed no detectable reduction in cell viability and outperformed nucleofection delivery by 60-fold [41]. Developments in NP strategies for delivery are discussed in a dedicated section below in more detail. Finally, Chin et al. (2013) used bisPNAs and 100 mer ssDNA donors to target the promoter of the human γ-globin gene in CD34+ cells [42]. While inactive in adults, specific mutations in the γ-globin promoter alter transcription factor binding and reduce silencing. Inducing such mutations are a strategy to alleviate the burden of hemoglobinopathies such as β-thalassemia and sickle cell disease. By nucleofection, Chin et al. demonstrated up to 1.63% editing using bisPNAs to introduce a -117 G→A γ-globin promoter mutation in cultured CD34+ cells [42]. Bolstered by improved nanoparticle-mediated delivery, bisPNA approaches substantially progressed PNA technologies towards viability as a potential therapeutic genetic technology. Still, rational chemical improvement of PNAs has the potential to optimize binding kinetics and enhance recombinogenic potency even further.

### 4.2. tcPNAs and First In Vivo Studies

To modulate the enthalpic component (ΔH) of PNA-DNA binding by strand invasion, tail-clamp (tc) PNA oligomers were designed by extending the PNA recognition domain on the WC face of the target DNA (Figure 2C [43,44]). This modification contributes to the binding enthalpy by increasing the number of base pairs (and H-bonds) annealing the PNA strand to its target [43,44]. For example, Bentin et al. showed that a 10-mer extension on the PNA C-terminus (which binds the DNA WC face) increased the thermal stability of the PNA-DNA complex ~ 40 °C and improves strand invasion into duplex DNA by up to 100-fold [43]. Interestingly, the improvement in binding efficacy for tcPNAs over bisPNAs is driven more by decelerated dissociation than by accelerated association, as demonstrated by data from independent groups showing tcPNAs to have two- to three-fold lower $k_{a}$ for strand invasion while also possessing much lower (> 250-fold) dissociation rate constants ($k_{d}$) relative to bisPNAs [43,44]. In this case, the direct relationship between $K_{D}$ and $k_{d}$ (eqn 2), and the direct relationship between $K_{D}$ and ΔG (eqn 3), have the cumulative effect of improving the strand invasion reaction by decreasing ΔG.

Equipped with the ability to form an overall more stable triplex, tcPNAs were first applied to gene editing by Schleifman et al. (2011) to introduce a stop codon into the CCR5 gene in human THP-1 cell lines and CD34+ cells [45]. As rationale behind this approach, naturally occurring knockout mutations of CCR5, a chemokine receptor required for HIV-1 entry into T-cells, imparts resistance to HIV infection. Nucleofected tcPNA reagents successfully modified 2.46% of THP-1 cells, a notable advantage over the 0.54% frequency achieved using bisPNAs in the same system. Modified cells demonstrated resistance to R5-tropic HIV-1 infection and were continually cultured for 98 days to demonstrate persistence. Five edited clones were maintained for 13 months with demonstrable heritable modification [45].

Schleifman et al. (2013) took this approach a step further marking the first ex vivo editing and engraftment studies utilizing PNA technologies encapsulated into PLGA NPs. In this study, PNA treated peripheral blood mononuclear cells (PBMCs) in culture were edited up to frequencies of 0.97% as determined by deep sequencing analysis [21]. The treated cell population was then engrafted into immune-deficient NOD-*scid IL2rγ^null^* mice. Flow cytometry analyses confirmed the presence of similar frequencies of engrafted human leukocyte and T-cell subsets in spleens 4 weeks post-transplantation for both treated and untreated PBMCs, indicating treatment had no effect on the ability of progenitor populations to engraft the animals. Finally, human PBMC engrafted mice were challenged with CCR5-tropic HIV-1_BaL_ virus by intraperitoneal injection. Engrafted mice treated with PNAs maintained higher overall CD4^+^ T-cell counts that rose to levels similar to uninfected mice with concordant viral RNA levels approaching undetected levels [21]. These experiments highlighted the combined advantages of improved tcPNA chemistry with an elongated WC-binding tail and optimized NP-mediated delivery. These basic features, with minor modifications, remain the standard for the most potent PNA editing approaches used today.

In vivo applications with optimized tcPNA and NP reagents emerged shortly after these developments were introduced. PLGA NPs were previously used for systemic delivery of drugs in FDA-approved applications. Thus, barriers to direct systemic administration were low as the same materials used in in vitro and ex vivo approaches can be safely introduced directly into the bloodstream of animals. The first in vivo application of PNA-mediated gene editing was described by McNeer et al. (2013) using tcPNAs encapsulated in various NP formulations. NOD-*scid IL2rγ^null^* mice engrafted with human CD34+ HSCs were treated by systemic injection with NP-delivered tcPNAs and donor DNA targeted to the CCR5 and β-globin loci [16]. Engrafted mice treated intravenously with tcPNA particles demonstrated detectable targeted CCR5 editing in bone marrow, spleen, thymus, gut, and lung. Deep sequencing of whole spleen revealed 0.43% total editing with possibly much higher editing (>14%) in some isolated human progenitor-derived colonies. To further demonstrate the ability of tcPNA to modify HSCs, the same authors transplanted marrow from tcPNA/DNA treated mice into untreated NOD-*scid IL2rγ^null^* mice [16]. CCR5 modification was noted in recipient mice 10 weeks after serial transplantation. Finally, this same approach was demonstrated using a different tcPNA targeted to a β-globin intron within a GFP fluorescent reporter in human HSCs engrafted mice. These results showed, for the first time, that PNA gene editing reagents can be delivered systemically to modify relevant targets in vivo.

NP delivered tcPNAs have successfully targeted airway epithelium in vivo. Fields et al. (2013) employed an optimized NP formulation for intranasal delivery of tcPNAs to target the previously described β-globin intron/GFP reporter in the lungs of mice [18]. The authors successfully edited lung epithelium as well as alveolar macrophages and alveolar epithelial cells. McNeer and Anandalingham et al. (2015) used this same approach to intranasally deliver tcPNA/DNA donors to target and correct the F508del mutation of the cystic fibrosis transmembrane conductance regulator (CFTR) gene in a mouse model [19]. This particular mutation is a prevalent pathologic cystic fibrosis mutation seen in patients. Corrective editing in intranasally treated mice was assayed by deep sequencing and using a functional nasal potential difference assay (NPD) that reports functional chloride efflux in vivo. Mice treated with tcPNA/DNA showed significant reductions in NPD readouts consistent with wild-type voltages and 5.7% correction of CFTR F508del mutation in lung epithelium [19]. The above studies signify an expansion of the target repertoire and means of delivery for in vivo application of therapeutic PNA gene editing. Again, rational design of PNAs and delivery reagents proved capable of improving potency and progressing the diverse applications of the system.

### 4.3. PNA Gamma(γ) Modification and Further In Vivo and In Utero Application

Recognizing the enhancements in strand invasion efficacy imparted to the PNA by extension of the WC binding domain (as in tcPNAs), groups have incorporated gamma(γ)-PNA residues (Figure 3B [29,46]) into this segment of a tcPNA to derive γtcPNA (Figure 2D [20]). Although this review will mostly emphasize the utility and superiority of this modification for gene editing applications [20,22], this strategy was based on much earlier work showing γ moieties improve recognition of single- and double-strand DNA targets, at least in the context of WC pairing by single, monomeric PNAs [47,48]. γPNAs possess a synthetically installed stereogenic center at the γ position of the PNA backbone (Figure 3B), and this modification induces global conformational selection in the entire PNA oligomer, resulting in helical preorganization in a manner determined by the stereochemistry of the γ-position [29,46]. With the appropriate γ-configuration, itself determined by selection of the appropriate amino acid enantiomer at the start of monomer synthesis, the PNA oligomer can be engineered to match the helicity of the DNA target [49].

Helical homology confers several intuitive and empirical improvements to the binding reaction. For example, γ-modification accelerates association and decelerates dissociation in the context of DNA binding [29], both of which converge to lower $K_{D}$ relative to unmodified PNA as shown by eqn 2. As predicted by eqn 3, the improved affinity (lower $K_{D}$) manifests as a stronger exergonic reaction for γPNA-DNA binding compared to corresponding reactions with PNA (−ΔΔG = 5 kJ/mol [29]). Also, the improvement in binding kinetics, particularly in the association phase, is especially salient in the context of strand invasion, since earlier reports have shown that, once formed, even hybrid complexes containing sub-optimal PNA designs and/or PNA-DNA combinations remain stable for extended periods [50], highlighting a critical role for an efficient initiation/nucleation step.

The free energy gain (ΔΔG) conferred by γ modification provides an opportunity to simplify the design criteria for PNA oligomers effective for strand invasion. Accordingly, Ly and coworkers have shown that strategic implementation of γ-modification can render clamp formation, as in bis- and tcPNAs, expendable in the context of strand invasion [47,48]. Single, monomeric PNAs of appropriate length (≥15 mer) have been modified with γ residues and shown to be effective for sequence-specific strand invasion, with corresponding displacement of the homologous region on the non-target strand [47,48]. There are two merits of this design simplification: (1) by decreasing the length of PNAs necessary for strand invasion, γPNA modifications engender significant reductions in synthetic costs and effort necessary to obtain useful reagents; (2) because the enthalpic component of the binding reaction does not require significant (or any) H-bonding on the HN face of a polypurine DNA target, but is instead provided by enhanced H-bonding on the WC face of even mixed targets, γPNAs expand the genomic targeting range beyond sites with pronounced asymmetry in the strand distribution of purines and pyrimidines. This latter consideration is especially salient for gene editing, which shows a systematic dependence of modification efficiency on triplex-target site separation, at least in the context of TFO-induced triplexes [51]. Appropriately modified monomeric γPNAs targeting mixed-sequence genomic sites therefore provide an opportunity to modulate the hybrid (PNA-DNA)-target site separation in a systematic, predictable manner to elicit gene editing (Figure 2E [17]).

The enhanced invasion ability and helical pre-organization of γPNAs have generated the most potent editing reagents to date enabling robust in vivo application. Bahal et al. (2014) used γ modified tcPNA co-encapsulated with ssDNA donors in NPs to edit mouse primary bone marrow cells ex vivo and in vivo and demonstrated their superiority to unmodified tcPNA reagents [17]. Using a similar β-thalassemia intron IVS2/eGFP reporter to previous studies, the authors demonstrated approximately fourfold higher editing frequencies when using γtcPNA over unmodified tcPNAs with identical sequences.

Bahal et al. (2016) followed-up these promising experiments by applying these same modified reagents to a β-thalassemia disease mouse model [20]. In this model, mice contain no alleles for murine β-globin and only a single copy of human β-globin containing a thalassemia-associated splicing mutation in the IVS2 intron (position 654 - T→C). Consequentially, mice demonstrate a marked β-thalassemia disease phenotype including microcytic anemia and splenomegaly. γtcPNA/ssDNA NPs designed to target the pathologic mutation were administered to animals systemically by intravenous injection. Treated animals demonstrated appreciable editing in target tissues such as bone marrow and γ modified tcPNA reagents outperformed both unmodified tcPNA and non-triplex-forming γ modified ssPNAs. Thus, these studies again demonstrate the advantageous properties of PNAs conferred by tail-clamp triplex forming moieties and γ modification. Moreover, the authors described impressive disease phenotype amelioration after NP administration. Treated animals demonstrated dramatic reductions in spleen size, reduced reticulocytosis, as well as a persistent, to at least 140 days, elevations in hemoglobin to wild-type ranges. Notably, intravenous treatments with NPs were not associated with any measurable increase in inflammatory cytokines [20].

Recent work demonstrated the proof-of-concept of in utero gene editing in a mammalian mouse model using NPs containing γtcPNA and donor DNA [22]. Stemming from the observation that hematopoietic stem and progenitor cells may be edited at a higher efficiency than already differentiated cells [16,20], Ricciardi et al. endeavored to achieve gene editing during fetal development, when an organism contains a higher proportion of dividing and expanding stem and progenitor cell populations [22]. Using a mouse model of β-thalassemia, the authors administered NPs containing γtcPNA and donor DNA to mice in utero via two administration routes: intraamniotic and intravenous via the vitelline vein. The procedure was safe: PLGA NP administration did not affect fetal plasma cytokine levels relative to controls. Moreover, PLGA NPs did not affect survival rates, weight gain, or gross anatomy of mice that had been treated in utero and successfully weaned. As intravenous delivery resulted in the greatest amount of NP accumulation in the fetal liver, the site of fetal hematopoiesis, the authors assessed phenotypic amelioration following intravenous NP administration. Hallmarks of disease pathology were reversed including underlying anemia, reticulocytosis, splenomegaly, and associated extramedullary hematopoiesis. Treated mice had 100% survival 500 days after in utero NP treatment [22].

Notably, Ricciardi et al. achieved an editing frequency of ~6% in total bone marrow and ~10% in hematopoietic progenitor cells after a single in utero NP treatment. Additionally, the in utero results were attained with a fraction of the dose of NPs used postnatally (185 μg versus 8 mg) [20,22]. The biology of a fetal stem cell may lend itself more readily to gene editing. RNA sequencing experiments suggest that fetal liver HSCs, in general, possess higher expression of DNA repair pathway-related genes than adult bone marrow HSCs. More specifically, KEGG pathway analysis found that genes related to mismatch repair, homologous recombination, nucleotide excision repair, and base excision repair pathways were more highly expressed in fetal liver HSCs when compared to adult HSCs [52].

In addition to a potentially favorable transcriptome, the population of fetal liver HSCs is rapidly expanding, with approximately 100% of HSCs cycling every 24 h, which is in stark contrast to the adult bone marrow HSCs that are 90–95% quiescent (reviewed in [53]). The ability to target or access this rapidly dividing stem cell population within the fetal liver with PNA/DNA NPs may account for the higher levels of gene editing observed prenatally and could represent an important therapeutic opportunity for site-specific gene editing of hematopoietic disorders before birth.

### 4.4. Ongoing Work and Democratization

Active research in the area of triplex PNA editing continues. Recent data shared by Piotrowski-Daspit et al. [54,55], Ricciardi et al. [56], and Quijano et al. [57] point to further in utero and in vivo applications of PNA technologies to models of cystic fibrosis, β-thalassemia, and sickle cell disease. Bolstered by novel approaches to NP delivery [54,55] and new insight into mechanistic underpinnings [58], PNA editing has strong potentially to develop even further in the near future. Meanwhile, using a similar approach to triplex editing, recent work by Fèlix et al. demonstrated the use of a new type of DNA-based triplex-forming molecule consisting of an elongated Watson–Crick binding region with a clamping anti-parallel Hoogsteen binding region [59]. These DNA oligos contrast single-stranded TFOs and are more similar in design to tcPNAs. Further, Watson–Crick binding DNA stretches featured the intended sequence modifications within the clamping molecule and thus did not require the introduction of a separate ssDNA donor. Authors described corrections of single point mutations in the adenosyl phosphoribosyl transferase (*aprt*) gene in CHO cells [59]. Newly emerging studies like these indicate exciting new directions for triplex editing as more groups begin to apply this conceptual approach. 

Notably, while PNA technologies have proven impressively effective, a limited number of groups are actively using these reagents for the purposes of gene editing. Required expertise in synthetic chemistry and novel design limit widespread use and ultimately the rate of development and improvement. Reminiscent of the slow uptake of initial nuclease-mediated gene editors, such as zinc-finger nucleases, it was not until the advent of TALENs, CRISPR, and democratized access to these technologies that use and understanding in the field began to accelerate. In the case of PNAs, resources are beginning to emerge that will enable their widespread adoption. A web tool designed to identify polypurine, and thus triplex-accommodating, regions within gene targets was developed by the Vazquez group allowing easy identifications of PNA-triplex targeting sites across human and mouse genomes (http://utw10685.utweb.utexas.edu/tfo/) [60]. Further, the advancement and adoption of automated peptide synthesizer platforms has begun to simplify and expedite a PNA synthesis process that previously required extensive and time-intensive chemistry protocols and expertise. Additionally, described below in Table 1, this review contains list of convenient guidelines and recommendations for the design and application of triplex-forming PNAs for gene editing. The development of PNAs into a clinically applicable and curative therapeutic tool depends on the ability of these novel molecules to be easily adopted and wielded by investigators. Reducing barriers to access and encouraging the adoption of PNAs for gene editing has promise to further accelerate advancement towards the ultimate goal of widespread therapeutic application in humans.

## 5. Delivery

As described above, employing various delivery strategies for PNA reagents rapidly accelerated the development of the technology for gene editing applications. Although PNAs hold significant therapeutic potential, on their own their biological application is limited by an inability to passively diffuse across cellular membranes. Using liposomes as a model for this hydrophobic barrier, Wittung et al. (1995) initially demonstrated that short PNAs (10 bp) had efflux times of 5.5 and 11 days, which was comparable to a control DNA [61]. With slow inherent uptake kinetics, adoption of successful delivery methods is pivotal to the in vitro and in vivo use of PNAs.

### 5.1. Peptide-Mediated Delivery of Peptide Nucleic Acids

Inspired by the uptake of TAT peptides [62,63,64], a class of well-known cell-penetrating peptide (CPP), PNAs have been directly modified with arginine residues to enhance their cellular uptake [29,65]. Zhou et al. synthesized PNAs bearing arginine sidechains at the alpha position and demonstrated enhanced perinuclear cellular uptake comparable to a fluorescently-labeled TAT peptide [66]. When this modification was moved to the gamma position, these uptake properties were retained, with the added benefit of preorganizing the PNA into a right-handed helix [67].

In addition to direct modifications, PNAs have also been conjugated to CPPs to enhance their cellular uptake [66,67,68]. CPPs are short, cationic peptides that vary in length, typically ranging from nine to thirty amino acids [62]. While several CPPs have been investigated to date, the most successful of these for delivery of PNAs has been penetratin, a sixteen residue peptide derived from the *Drosophila Antennapedia* gene [69,70,71]. Using PNAs targeted against the HIV trans-activation response (TAR) element (an RNA stem-loop), Turner et al. demonstrated that PNAs conjugated to penetratin could potently inhibit Tat-dependent *trans*-activation in HeLa cells [70]. Interestingly, when coupled to a TAT peptide, the PNAs were completely inactive, unless accompanied by the addition of chloroquine, which was presumed to facilitate endosomal escape [70].

Similarly, Rogers et al. (2012) have previously employed penetratin to deliver PNAs in vitro and in vivo [72]. Using PNAs targeted against a chromosomally integrated *supFG1* reporter gene in mouse cells, they showed that penetratin-PNA conjugates achieved higher levels of uptake and targeted mutagenesis than passive uptake of the PNA alone. Importantly, these penetratin-PNA conjugates demonstrated enhanced biodistribution and targeted genome modification in vivo in several somatic tissues and compartments of the hematopoietic system [72].

In addition to traditional CPPs, recent work has described a strategy to deliver PNAs using a peptide which preferentially accumulates in acidic microenvironments [73,74]. Under physiologic pH, pHLIP (pH (low) insertion peptide)), weakly associates with the cellular membrane [75]. As pH is reduced, pHLIP becomes protonated, resulting in a transmembrane α-helix with its C-terminus inserted across the cellular membrane [76]. Using this peptide, Cheng et al. (2015) delivered PNAs against oncomiR-155, resulting in significant delays in tumor growth in vivo [77].

While peptide-mediated delivery of PNAs remains a promising approach for in vitro and in vivo applications, the need for excessively high and repeated dosing to achieve therapeutic benefit (10–50 mg/kg) reduces the potential for clinical translation. The broad effectiveness of CPPs specifically as delivery agents is also limited by their endosomal entrapment [78], requiring the use of lysosomotropic agents such as chloroquine or Ca^2+^ to enhance effectiveness [70,79]. pHLIP, though useful for targeting acidic microenvironments, is not a viable strategy for delivering PNAs to non-acidic tissues. Moreover, we have recently shown that pHLIP may be less effective at delivering PNAs greater than 25 nucleotides in length [75]. Finally, while peptides may serve as effective delivery vehicles for PNAs, the need for co-delivery of donor DNA for gene editing would require additional conjugation strategies to deliver both molecules. To overcome these limitations, recent work with PNAs has focused on the development of polymeric nanoparticles (NPs) to efficiently entrap and deliver PNA as well as donor DNA molecules to cells in vitro and in vivo.

### 5.2. PLGA Nanoparticle-Mediated Delivery of PNAs

As an alternative to peptide-mediated delivery of PNAs, several groups have investigated the use of NPs. While many of these delivery systems have been reviewed elsewhere [80], efforts have focused on developing NPs from poly(lactic-co-glycolic acid) (PLGA), a biocompatible and biodegradable polymer, well-known to the FDA [81]. In addition to their favorable safety profiles [82,83], drug release from PLGA NPs is highly tunable by adjusting polymer molecular weight and ratio of lactic to glycolic acid [84]. Recently, Mandl et al. showed that NP size can be tuned to alter biodistribution and accumulation of NPs in sites of interest, such bone marrow or lung [85].

In 2011, McNeer et al. first demonstrated that PNA and donor DNA molecules could be formulated into PLGA NPs using a double-emulsion solvent evaporation technique. In this process, the PNA and donor DNA encapsulants are first mixed and subsequently added to the polymer under vortex, forming the first water-in-oil emulsion. Following sonication, the PLGA NPs are stabilized through dropwise addition into a second aqueous solution containing a surfactant. After a final sonication step, the NPs are flash frozen and lyophilized prior to use. By co-encapsulating these molecules into NPs, this approach was used to mediate recombination in human CD34+ cells, resulting in site-specific modification of the β-globin locus [41]. While the level of modification was relatively modest (0.5–1%), the improvements in cell viability were significant compared to standard electroporation, which reduced cell viability to ~20% after 24 h. This observation was in sharp contrast to cells treated with blank or PNA/DNA NPs, which demonstrated 100% cell viability [41]. The use of PLGA NPs loaded with PNA and donor DNA resulted in a 60-fold increase in editing compared to cells electroporated with these reagents.

While robust in its delivery of PNA and donor DNA molecules in vitro, a major benefit of using PLGA NPs is their ability to effectively deliver these molecules in vivo [86]. Based on previously discussed work modifying CCR5 [41], McNeer, Schleifman et. al, used NPs with surface coated with TAT or penetratin peptides to treat mice engrafted with human CD34+ cells [16]. While prior work had demonstrated that TAT conjugated PNAs were not effective in the absence of chloroquine [63], TAT conjugated NPs consistently produced higher levels of gene editing in vitro in human CD34+ cells [16]. When administered systemically, TAT modified NPs achieved CCR5 modification in multiple tissues including disease relevant CD4+ and CD34+ human cells [16]. Importantly, these NPs were non-toxic to bone marrow or spleen progenitors as demonstrated by colony forming assays. Beyond these initial experiments PLGA NPs have been extensively used to deliver PNA and donor DNA. As discussed in above sections, PLGA NP delivery has been successfully applied to multiple in vivo studies and demonstrated the ability to targeted native HSCs in mice with therapeutic effects in disease models. Notably, and consistent with prior work, Bahal et al. (2016) and Ricciardi et al. (2018) found PNA/DNA PLGA NPs did not induce inflammatory cytokine secretion in treated animals [20,22].

### 5.3. PBAE/PLGA/MPG Nanoparticle-Mediated Delivery of PNAs

While the use of PLGA has been extensively explored for the delivery of PNA and donor DNA, new materials are being investigated, including polymer blends of PLGA with poly(β-amino esters) (PBAE) [18,19,87]. In particular, NPs made from blends of PLGA and PBAE have shown exceptional promise for the delivery of PNA and donor DNA to lungs following intranasal administration [18,29]. In developing these NPs, Fields et al. carefully explored the blending of PLGA with PBAE to enhance NP transfection and loading of DNA [88,89], while attenuating the observed toxicity of this cationic polymer [87]. To further enhance uptake, these NPs were surface-coated with a DSPE-PEG lipids conjugated to CPPs. With these modifications, cellular uptake of NPs in vitro was substantially improved [87] and subsequently shown to enhance NP uptake in mice following intranasal administration [18]. In particular, NPs were found to associate with approximately 30% of all lung cells, in contrast to unmodified NPs, which demonstrated little to no cellular associated with lung [18]. Though levels of editing were modest (0.6% in alveolar macrophages and 0.3% in alveolar epithelial cells), this early work provided proof that this approach could be used to edit genes in the lung following intranasal NP administration. Further, as with PLGA NPs, treatment with PLGA/PBAE/MPG NPs did not increase inflammatory cytokine production following in vivo application [19].

## 6. Mechanisms of PNA-Mediated Gene Editing

### 6.1. PNA Triplex Repair and Recombination

PNA-mediated gene editing relies on the ability of endogenous cellular factors to detect anomalous PNA/DNA structures within the genome. Structure repair ultimately enables a recombination event with a nearby homologous ssDNA donor molecule to incorporate a permanent sequence modification. The mechanisms by which PNA detection and repair can instigate recombination are not yet fully elucidated. Thus far, PNA editing has been shown to depend, in part, on nucleotide excision repair (NER) [15,40]. NER is a non-mutagenic repair pathway responsible for the removal of bulky, helix-distorting lesions within DNA—an effect known to be imparted by PNA invasion and structure formation [8,88]. NER relies on a network of factors including context-specific recognition factors (XPC, CSA/CSB, XPA/RPA), specialized helicases (XPD, XPB), and endonucleases that generate single strand nicks (XPG, XPF). Xeroderma pigmentosum group A (XPA), a central NER factor responsible for lesion detection and factor assembly, has been shown to be important for PNA editing to occur. Experiments in XPA-depleted cell-free HeLa extracts by Rogers et al. (2002) showed marked reductions in PNA repair and recombination [15]. Chin et al. (2008) made a similar observation when XPA was knocked down by siRNA in a human K562 cell line [40]. Notably, no other targeted gene editing technology is known to utilize non-mutagenic NER pathways. Rather, nucleases such as Cas9, TALENs, and zinc-finger nucleases are known to feature homology directed repair (HDR) or mutagenic non-homologous end joining (NHEJ) pathways at double strand breaks with mechanistically unknown contributions from Fanconi Anemia repair factors [89,90]. Base Editor and deaminase approaches, meanwhile, predominantly rely on enzymatic deamination of nucleobases, base excision repair (BER), and mismatch repair (MMR) to achieve single base modifications [91].

### 6.2. TFO Triplex Repair and Recombination

While the repair and recombination mechanisms specific to PNA-mediated editing remain under investigation, earlier work on TFO-mediated recombination offers insights into other structure-induced recombination processes and how PNA editing may occur. Similar to PNA triplexes, NER seems to play an important role in TFO triplex repair and recombination. Experiments in human XPA-immunodepleted cell-free extracts [26] and in XPA knockout human fibroblasts [14] both show marked reductions in TFO repair and recombination. Plasmid-based experiments in mammalian cells by Faruqi et al. (1999) showed reduced TFO-mediated recombination in XPA, XPG, and XPF deficient cells—an effect that was rescued partially by XPA cDNA complementation. Interestingly, knockdown of MMR factors MSH2 and MLH1 did not have an effect on overall recombination efficiency [14].

### 6.3. Endogenous Triplex Repair and Recombination

Insights from how cells repair endogenous triplex structures reveal more potential mechanisms of triplex repair and resolution. In work recently published by the Vasquez group (2018) they describe replication-dependent and replication-independent pathways for the repair of DNA triplexes (H-DNA) that form spontaneously between gDNA strands [3]. While replication-independent repair and cleavage rely on NER factors XPG and XPF, flap endonuclease 1 (FEN1) is a key factor that binds and cleaves endogenous H-DNA during replication [3]. The role of FEN1 in PNA or TFO-mediated recombination has yet to be explored.

### 6.4. ssDNA Donor Recombination and Rad51

Rad51 recombinase, an HDR factor that binds ssDNA overhangs for homology search and invasion, appears to play a role in TFO-mediated recombination. In the case of TFOs tethered to ssDNA templates, Rad51 overexpression increases targeted recombination with an episomal vector [26]. Conversely, experiments with whole-cell extracts immunodepleted of Rad51 showed reduced recombination frequencies [26].

In the broader field of gene editing, however, the exact role Rad51 plays in recombination with ssDNA donors remains unclear. Interestingly, work from the Corn group (2018) in CRISPR/Cas9 systems shows that while Rad51 siRNA knockdown significantly reduced recombination frequencies when using dsDNA donors (plasmid), no such effect existed when using ssDNA donor molecules [90]. Complicating the picture further, work from Davis and Maizels demonstrated that when using single strand nickase mutants of Cas9 and a ssDNA donor, thus leaving one DNA strand intact, knockdown of Rad51 improved recombination frequencies eightfold [92]. The same effect was observed when BRCA2, an HDR factor directly upstream of Rad51, was knocked down as well [92]. The authors go on to suggest that a distinct HDR sub-pathway called single strand annealing (SSA) and factors such as Rad52 may be responsible for ssDNA incorporation in the presence of single stranded nick intermediates [92,93].

In summary, Rad51 may promote, antagonize, or have little effect on ssDNA donor incorporation, depending on the context and approach. These diverging results are likely explained by how ssDNA oligonucleotides are able to interact with the intermediate recombinogenic structures or breaks induced by gene editing technologies. The ecosystem of often competing repair factors associated with structures at these genomic locations dictate preferred pathways of repair and recombination. Ultimately, future experiments will be needed in order to clarify the role Rad51 and strand search HDR pathways play in gene editing with ssDNA donors. Rad51 contribution to PNA-mediated editing is currently under study.

## 7. Other PNAs for Gene Editing

### 7.1. pcPNAs for Gene Editing

An alternative strategy to improve the exergonicity of strand invasion using monomeric PNAs has been explored by designing PNA pairs to hybridize both strands of the target region (Figure 2F [94]). This strategy relies largely on reaction enthalpy to drive hybridization, by effectively doubling the number of base pairs in the thermodynamic product of binding relative to the starting duplex but imposes a significant penalty in translational entropy since strand invasion in this context is a tetramolecular reaction. However, successful strand invasion with the appropriate combination of monomeric PNAs [94] is clear evidence that upon DNA hybridization, enthalpic gain compensates for entropic loss (ΔH<TΔS), as is often observed for DNA-ligand interactions [95]. Crucial for the effectiveness of this strategy is nucleobase modification to increase enthalpic contribution while preventing self-quenching of the PNA pair, which would otherwise be WC complementary. Specifically, the diaminopurine (Dap) nucleobase has been substituted for adenine (A) to provide an additional H bond donor for pairing with thymine (T) (Figure 3C [94]). The more stable Dap:T pair relative to A:T pair, coupled with the expanded surface area of Dap that stabilizes base pair stacking interactions, drives exergonicity by lowering ΔH. Furthermore, to prevent quenching of the PNA pair, thiouracil (sU) has been substituted for T (Figure 3C [94]), a modification which, although inducing mild destabilization of the PNA-DNA hybrid, abrogates any pairing between the modified nucleobases (Dap:sU) due to increased electron density (and repulsion) relative to Dap:T and A:sU (Figure 3C).

Applying these principles, so-called pseudo-complementary PNAs (pcPNAs) were successfully applied to gene editing in 2009 by Lonkar et al. using a cell culture model [96]. CHO cells containing the GFP-IVS2-1 β-thalassemia reporter, described previously in this review, were electroporated with a pair of opposite strand-binding pcPNAs and ssDNA donor. FACS analysis of treated conditions demonstrated up to 0.78% correctly edited, GFP+ cells [96]. Thus, pcPNAs are capable of inducing targeted recombination with a ssDNA template, likely in a manner distinct from triplex-forming PNAs. Few studies have followed up this approach to editing since this study. Future work with these reagents, however, may provide further insights into the breadth of structures capable of inducing recombination for gene editing. While there is some evidence that NER pathways may also contribute to pcPNA-mediated editing [96], how this overall mechanism compares to triplex forming PNAs remains uninvestigated.

### 7.2. ssPNAs for Gene Editing

Another approach, reported by Bertoni and colleagues, features the use of a single stranded WC-binding PNA (ssPNA) without a ssDNA donor [97,98]. This strategy uses ssPNAs designed to bind a mutation target within the genome forming a PNA/DNA heteroduplex opposite a displaced gDNA strand. ssPNAs invade gDNA helices to target a single strand of DNA directly by WC pairing, and thus have no sequence restriction and do not need to accommodate Hoogsteen binding. These molecules are designed as ~25 mers homologous to a coding or template strand target with the exception of the intended modified base. Initial work by Kayali et al. in 2014 used ssPNAs to correct a splicing mutation in the dystrophin gene characteristic of the disorder Duchenne muscular dystrophy (DMD) [97]. Transfection of anti-template strand and anti-coding strand 18 mer ssPNAs via Lipofectamine™ 2000 into myoblasts derived from a DMD mouse model resulted in ~3% and ~7% editing respectively. Authors also described dystrophin-positive muscle fibers in DMD mice after direct injection of free ssPNAs directly into the tibialis anterior muscle. Two weeks after treatment, qPCR methods determined 2.8% of template strand and 3.3% of coding strand targeted alleles to be edited. Dystrophin-positive edited fibers were detected in vivo four months after treatment [97]. Further studies from the same group determined these reagents were capable of editing satellite cells ex vivo from the DMD mouse model and that modified cells were capable of self-renewal and differentiation [98]. Engrafted satellite cells in DMD mice differentiated and persisted at least 24 weeks. Notably, the frequency of dystrophin-positive muscle fibers increased with time and this effect correlated with improved muscle morphology in the tibialis anterior of mice [98].

Studies featuring the use of ssPNAs are of considerable interest not only because they expand the potential therapeutic repertoire of PNA technologies but because of their presumably distinct mechanisms of action. Taken together, these results suggest the exciting possibility that polymerases may be able to use PNAs as a viable substrate to introduce deoxynucleotides opposite PNA nucleobases. In this particular case, one could imagine MMR and BER pathways of repair implicated in processing single base mismatches within novel PNA/DNA duplex structures. Similar to PNA triplex-mediated editing, however, more work concerning the repair mechanisms by which these structures are processed is needed to further understand and rationally improve this approach.

## 8. Perspectives and Limitations

PNA reagents are a conceptually distinct approach to gene editing. Rather than relying on the catalytic activity of enzymes on genomic DNA substrates, PNAs non-enzymatically generate unusual nucleic acid structures within the genome to promote recombination. As a result of this novel approach, preliminary studies suggest the repair mechanisms by which PNAs accomplish gene editing is also distinct. In fact, PNA editing may utilize inherently non-mutagenic pathways such as nucleotide excision repair (NER), offering a powerful advantage over technologies like CRISPR/Cas9 that are characteristically plagued by mutagenic repair outcomes [15,40,89].

PNA-mediated gene editing has progressed rapidly over the past two decades. Advances in optimized PNA chemistry and binding kinetics as well as sophisticated non-toxic means of delivery are responsible for propelling the applications of PNA editing reagents forward. Since initial observations of induced recombination using plasmid systems in cell-free extracts, PNAs have evolved into potent tools capable of ex vivo, in vivo, and in utero application in mice with therapeutically consequential effects [16,17,18,19,20,21,22]. Despite impressive progress informed by the chemical and biomedical engineering aspects of PNA technologies, however, approaches to advancement driven by biological, mechanistic insight remain mostly untapped.

Understanding the mechanisms of PNA structure recognition, repair, and recombination hold enormous promise for the future of the technology and more broadly in the rapidly developing field of gene editing. While improvements to PNA chemistry have substantially progressed editing potency over time, a detailed understanding of how these modifications are preferentially detected within the genome and lead to or improve recombination with ssDNA donors remains elusive. Ultimately, a truly rational approach to reagent design must be informed by the mechanistic underpinnings of PNA-induced repair and recombination. This information has the capacity to further inform PNA reagent chemistry and design as well as provide opportunities to further improve potency by biasing relevant pathways of repair. Moreover, investigating the likely novel means by which structure-specific repair interfaces with homologous recombination may reveal pathways to non-mutagenic gene editing not previously considered.

## Figures and Tables

**Table 1:** Guidelines for Triplex PNA Design for Gene Editing

Guidelines for Triplex PNA Design for Gene Editing
Target polypurine (A or G) sequence stretches in proximity to modification of interest, ideally ≥ 7 consecutive bases and within 500 bp of intended edit
PNA design: ≥20 Watson–Crick base stretch followed by flexible linker sequence and antiparallel triplex-forming Hoogsteen base stretch corresponding to polypurine target, three lysine residue cap at each N and C-terminus
PNA modification: consider introduction of gamma (γ) modified PNA monomers distributed throughout sequence
ssDNA donor: 60 mer single-stranded DNA oligonucleotide with centered modification sequence and three terminal phosphorothioate backbone modifications on 3′ and 5′ ends
Nanoparticle encapsulation at 2:1 PNA:DNA ratio
If possible, screen multiple candidate PNA target sequences and modification approaches for optimal editing activity
